# Immune Tolerance to Apoptotic Self Is Mediated Primarily by Regulatory B1a Cells

**DOI:** 10.3389/fimmu.2017.01952

**Published:** 2018-01-19

**Authors:** Katherine Miles, Joanne Simpson, Sheila Brown, Graeme Cowan, David Gray, Mohini Gray

**Affiliations:** ^1^MRC Centre for Inflammation Research, Queen’s Medical Research Institute, University of Edinburgh, Edinburgh, United Kingdom; ^2^School of Biological Sciences, Institute of Immunology and Infection Research, Ashworth Laboratories, The University of Edinburgh, Edinburgh, United Kingdom

**Keywords:** regulatory, B cell, B1a, apoptotic cell, immune tolerance

## Abstract

The chronic autoimmune inflammatory diseases, systemic lupus erythematosus and Sjogren’s syndrome, develop when tolerance to apoptotic cells (ACs) is lost. We have previously reported that this tolerance is maintained by innate-like, IL-10 secreting regulatory B cells. Two questions remained. First, do these regulatory B cells belong predominantly to a single subset of steady-state B cells and second, what is their specificity? We report here that innate-like B cells with markers characteristic for B1a cells (CD43^+ve^CD19^hi^CD5^+ve^IgM^hi^IgD^lo^) constitute 80% of splenic and 96% of peritoneal B cells that respond to ACs by secreting IL-10. AC responsive B1a cells secrete self-reactive natural antibodies (NAbs) and IL-10, which is augmented by toll-like receptor (TLR) 7 or TLR9 stimulation. In so doing, they both accelerate the clearance of dying cells by macrophages and inhibit their potential to mount proinflammatory immune responses. While B1a cells make prolonged contact with ACs, they do not require TIM1 or complement to mediate their regulatory function. In an animal model of neural inflammation (experimental autoimmune encephalomyelitis), just 10^5^ activated B1a B cells was sufficient to restrain inflammation. Activated B1a B cells also induced antigen-specific T cells to secrete IL-10. Hence, regulatory B1a cells specifically recognize and augment tolerance to apoptotic self *via* IL-10 and NAbs; but once activated, can also prevent autoimmune mediated inflammation.

## Introduction

Some years ago, Wolf et al. noted that B cell-deficient mice develop a chronic form of neural inflammation called experimental autoimmune encephalomyelitis (EAE) (1), which was later attributed to the loss of B cell IL-10 production (2). IL-10 is a broad spectrum anti-inflammatory cytokine that potently suppresses the activation of macrophages and dendritic cells (3, 4). Further studies have failed to identify a specific subset of IL-10 secreting regulatory B cells that express a particular transcription factor, akin to Foxp3 regulatory T cells (5–7). Instead, B cells with a capacity to regulate immune responses *via* IL-10 secretion have been described among activated B cells that express the surface markers CD5 and CD1d (8, 9), T2-marginal zone precursor B cells (10, 11), and plasma cells (12, 13).

Our own focus has been to understand whether regulatory B cells play a role in preventing a breakdown in tolerance to apoptotic cells (ACs) (7, 14, 15), the loss of which leads to autoimmune rheumatic diseases, including systemic lupus erythematosus (SLE), Sjogren’s syndrome, and systemic sclerosis (16). Following programmed cell death, ACs express immunogenic intracellular (IC) self-antigens on their cell surface (17–19). The mechanism for maintaining tolerance to apoptotic self is believed to rely almost exclusively on their rapid clearance by phagocytes (20, 21), which is accelerated by polyreactive natural antibodies (NAbs) that bind to AC expressed neoantigens (22). While central and peripheral tolerance mechanisms also purge many self-reactive B and T cells; a population of innate-like B cells, within the marginal zone (MZB) and B1a subsets, are selected on their ability to respond to self, developing normally even in the absence of foreign antigenic stimulation (23, 24). B1a cells are a major source of IL-10 (25), inhibiting the progression of both innate and adaptive immune responses, preventing tissue damage, but at the cost of impeding pathogen clearance (26). The presence of self-reactive innate-like B cells is not normally associated with autoimmunity, in spite of their frequent exposure to ACs in secondary lymphoid organs and sites of inflammation. Conversely, B1a B cells are also known as essential first responders to pathogens in the lung and gut, secreting proinflammatory GM-CSF (24, 27–29). Thus, a mechanism to ensure that ACs are sensed as tolerogenic by innate-like B cells is likely to be important.

We have previously reported, that splenic CD21^hi^CD23^low^ B cells and CD5^+ve^ peritoneal B cells can be activated by antigen-specific T cells (*via* CD40) or directly *via* toll-like receptor (TLR) ligands to secrete IL-10 in response to ACs. *In vivo*, mice given ACs at the time of inducing collagen-induced arthritis or EAE are protected from inflammation, generating lower titers of auto-antibodies, along with an increase in antigen-specific IL-10 secreting T cells (14). The B cells sense AC expressed DNase-sensitive determinants *via* their BCR and make IL-10 in a TLR9-dependent process (15). However, we do not know which particular antigens self-reactive regulatory B cells recognize or whether immune regulatory function is mediated predominantly by a particular subset of B cells.

This study addressed those questions and identified that 80% of splenic and 96% of peritoneal, AC responsive, innate-like regulatory B cells were B1a B cells. AC responsive regulatory B cells (ACBregs) make prolonged contact with ACs and secrete both IL-10 and self-reactive NAbs. This both enhances AC clearance and likely prevents a breach in self-tolerance. Additionally, they induce naive T cells to secrete IL-10, but they do not require the expression of Tim1 or C1q to exert their regulatory function.

## Materials and Methods

### Mice

IL-10-GFP, TIM1^−/−^ C57BL/6 (see Figure S2Cii in Supplementary Material for assessment of genotype), TIM1^−/−^ BALB/c (see Figure S2Ci in Supplementary Material for assessment of genotype), C1q^−/−^, complement receptor 2^−/−^ (CR2^−/−^) (see Figure S2J in Supplementary Material for confirmation of phenotype), DO11.10 TcR Tg mice, and OTII-Ly5.1 TcR Tg mice (both OVA_323–339_ peptide specific) were bred and maintained under specific pathogen free conditions in the Animal Facilities at the University of Edinburgh, UK. IL-10-GFP mice were kindly provided by Dr. Richard Flavell (Yale University, New Haven, CT, USA), TIM1^−/−^ mice by Prof. Andrew McKenzie (Cambridge, UK), C1q^−/−^ mice by Prof. M. Botto (Imperial) and CR2^−/−^ mice by Prof. Kevin Marchbank (Newcastle). Wild-type (WT) C57BL/6 and BALB/c mice were bred in house. Mice were used at 8–12 weeks of age and were sex and age matched. All experiments were covered by a Project License granted by the Home Office under the Animal (Scientific Procedures) Act 1986. Locally, this license was approved by the University of Edinburgh Ethical Review Committee.

### Flow Cytometry and FACS Sorting

**Figure 1:**
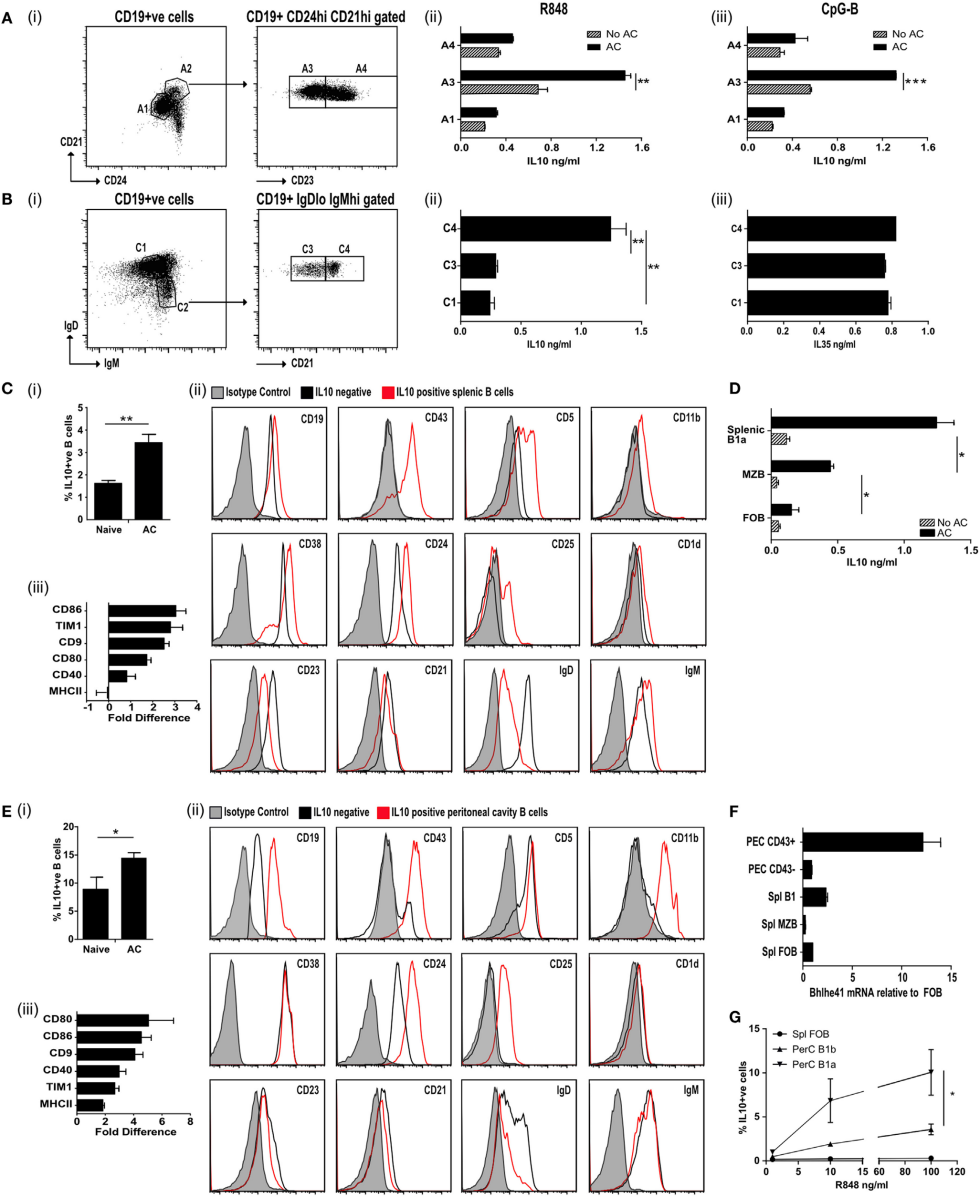
IL-10^+ve^ apoptotic cell (AC) responsive B cells express CD43^+^CD24^hi^CD21^hi^CD23^lo^IgD^lo^IgM^hi^. **(A)** Splenic CD19^+ve^ cells were FACS sorted (i) into CD24^lo^CD21^lo^ (A1; 69.8% of all B cells) and CD24^hi^CD21^hi^ (A2; 9.8% of all B cells). A2 was further sorted into CD23^lo^ (A3 50.8% of A2) and CD23^hi^ (A4 48.9% of A2). B cells from A1, A3, and A4 were activated through TLR7/8 (with R848) (ii) or TLR9 (with CpGB) (iii) with apoptotic cells (solid bars) or without apoptotic cells (patterned bars). IL-10 was measured in the supernatants after 72 h. See Figure S1A in Supplementary Material for cell purity. **(B)** Splenic CD19^+ve^ cells were FACS sorted (i) into IgD^hi^IgM^lo^ (C1 52.5% of all B cells) and IgD^lo^IgM^hi^ (C2 13.1% of all B cells). C2 was further sorted into CD21^lo^ (C3 49.7% of C2) and CD21^hi^ (C4 46.5% of C2). C1, C3, and C4 were cultured in the presence of R848 with apoptotic cells. IL-10 (ii) and IL-35 (iii) was measured in the supernatants after 72 h. See Figure S1B in Supplementary Material for cell purity. **(C)** Apoptotic cells were injected IV on D0, 2, and 5 into IL-10-GFP expressing mice. Splenic CD19^+ve^ cells were isolated on D7. (i) IL-10^+ve^ B cells. (ii) Surface markers of IL-10-GFP^+ve^ B cells (in red) and IL-10-GFP^−ve^ B cells (in black) compared to isotype control (in shaded gray). (iii) The fold difference in mean florescence intensity (MFI) (IL-10-GFP^+ve^ versus IL-10-GFP^−ve^ B cells), is shown for a range of other surface markers. See Figure S1C in Supplementary Material for sorting strategy and cell purity. **(D)** CD19^+ve^ B cells were sorted into MZB (CD19^+ve^CD23^lo^CD21^hi^CD43^−ve^), B1a (CD19^+ve^CD23^lo^CD21^hi^CD43^+ve^) B cells, and follicular B (FOB) cells (CD19^+ve^CD23^+ve^CD21^lo^CD43^−ve^). Cells were cultured with 2 μg/ml OVA peptide, DO11.10 CD4^+^ T cells ± apoptotic cells. After 72 h cells, the total IL-10 secreted by stimulated B and T cells was measured by ELISA. **(E)** Apoptotic cells were injected IV on D0, 2, and 5 into IL-10-GFP expressing mice. Peritoneal cavity CD19^+ve^ cells were isolated on D7. (i) IL-10^+ve^ B cells. (ii) Surface markers of IL-10-GFP^+ve^ B cells (in red) and IL-10-GFP^−ve^ B cells (in black) compared to isotype control (in shaded gray). (iii) The fold difference in MFI (IL-10-GFP^+ve^ versus IL-10-GFP^−ve^ B cells), is shown for a range of other surface markers. **(F)** CD43^+ve^ splenic and peritoneal B1 B cells express higher levels of transcription factor Bhlhe41, when compared to splenic FOB and marginal zone B cells or peritoneal CD43^−ve^ B cells. Data were normalized using 18S then expressed relative to FOB. **(G)** B cell subsets were sorted into splenic FOB (CD23^hi^CD21^lo^CD43^−ve^), peritoneal cavity B1a (CD19^hi^CD43^+ve^CD5^+ve^) and B1b (CD19^hi^CD43^+ve^CD5^−ve^) cells. Sorted cells were cultured in the presence of apoptotic cells and R848 at varying concentrations. Cells were stained after 72 h for intracellular CD19^+ve^ IL-10. All experiments are representative of three individual experiments [except **(C,D)**, where FACs data are representative of 13 individual experiments. Activation marker changes are graphed from pooled data of min six individual mice]. Statistical differences were determine by unpaired Student’s *t*-test *P < 0.05, **P < 0.01, and ***P < 0.001.

**Figure 2:**
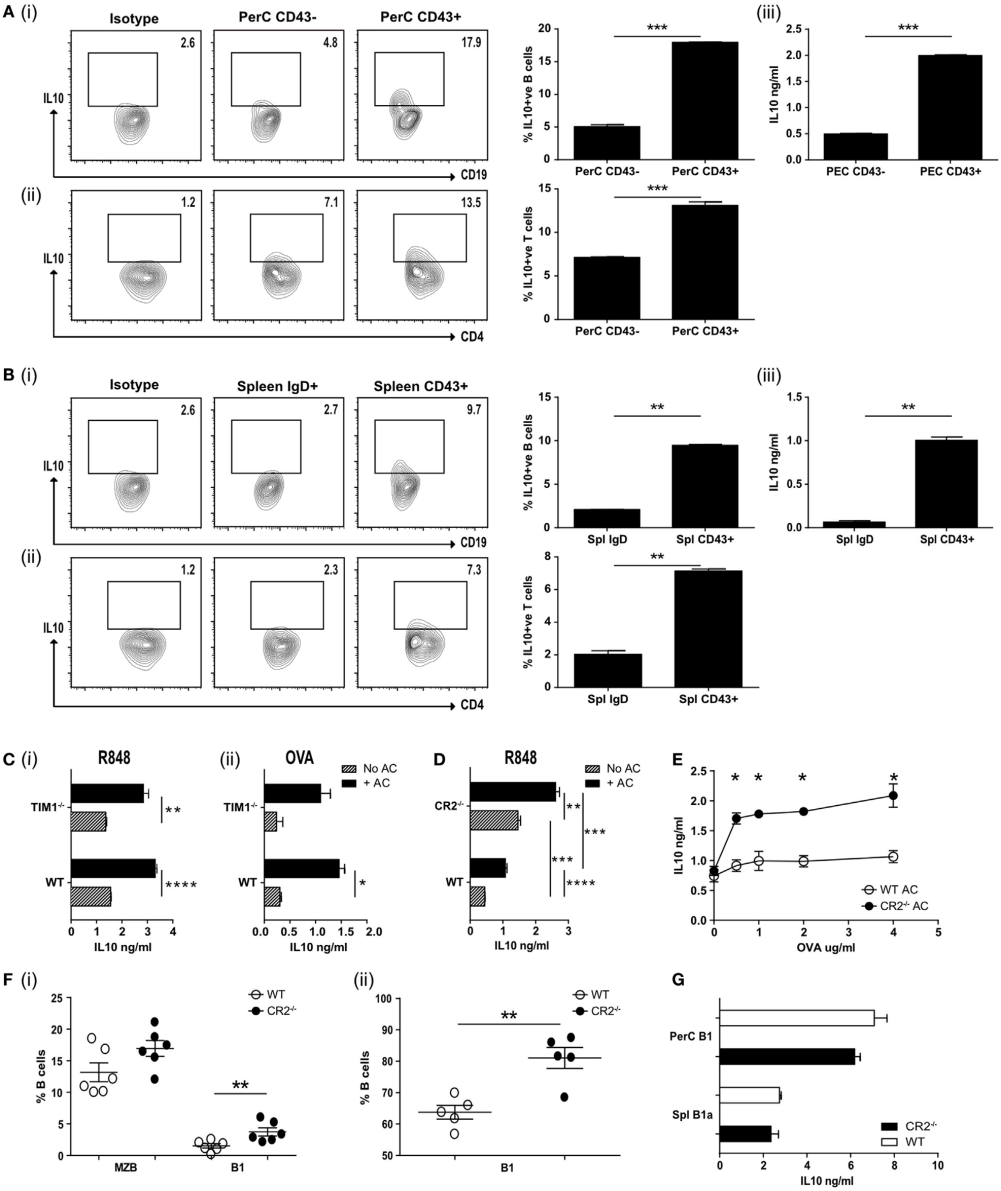
**(A)** Apoptotic cells were injected IV at the time of OVA-complete Freund’s adjuvant (CFA) antigenic challenge *in vivo*. Peritoneal CD19^+ve^ cells were harvested on D7 and FACS sorted into CD43^−ve^ or CD43^+ve^, cultured with OVA peptide and OVA-specific T cells and IL-10 measured after 72 h. Representative flow plots and pooled data for intracellular IL-10 staining are shown for CD19^+ve^ cells (i) and CD4^+ve^ cells (ii). Secreted IL-10 was measured in the supernatants by ELISA (iii). Cell purity and CD19 expression can be found in Figure S2A in Supplementary Material (*N* = 10 mice). **(B)** Splenic CD19^+ve^ cells from OVA-CFA challenge experiment in **(A)** were FACS sorted into IgD^hi^ follicular B (FOB) cells and IgD^lo^CD24^hi^CD43^+ve^ B1a cells. Sorted cells were cultured with OVA peptide and OVA-specific T cells and IL-10 measured after 72 h. Representative flow plots and pooled data for intracellular IL-10 staining are shown for CD19^+ve^ cells (i) and CD4^+ve^ cells (ii). Secreted IL-10 was measured in the supernatants by ELISA (iii). Cell purity and CD19 expression can be found in Figure S2B in Supplementary Material (*N* = 10 mice). **(C)** wild-type (WT) BALB/c and TIM1^−/−^ BALB/c B cells (IgD^lo^IgM^hi^CD21^hi^) were cultured with (black bars) and without (patterned bars) apoptotic cells and activated with R848 (i), and OVA plus OVA-specific T cells (ii). IL-10 in the culture supernatants was assessed after 72 h (*n* = 8). **(D)** CD19^+ve^IgD^lo^IgM^hi^ B cells were FACs sorted from complement receptor 2^−/−^ (CR2^−/−^) and control C57BL/6 mice. Cells were cultured with (solid black bars) and without (patterned bars) apoptotic cells in the presence of R848. IL-10 was measured in the culture supernatants after 72 h. Data are representative of two experiments using 10 mice in total. **(E)** Splenocytes from mice immunized with OVA-CFA and given apoptotic cells were harvested on D7 and re-stimulated with OVA. IL-10 was measured in the supernatants after 72 h. WT controls are shown with open circles, complement receptor 2^−/−^ (CR2^−/−^) mice with filled circles. Data pooled from three individual mice, representative of two experiments, five mice total. **(F)** Spleen and peritoneal cavity CD19^+ve^ B cells were harvested from naive C57BL/6 and CR2^−/−^ mice. Cells were stained to determine subsets of splenic (i) MZB (IgD^lo^IgM^hi^CD43^−ve^), B1 (IgD^lo^IgM^hi^CD43^+ve^) or peritoneal cavity (ii) B1 (CD19^+ve^CD43^+ve^). WT are shown with open circles and CR2^−/−^ with filled circles. *N* = 6 (spleen) or 5 (peritoneal cavity). **(G)** Splenic (CD19^+^IgD^lo^IgM^hi^CD43^+^) and peritoneal (CD19^hi^CD43^+^) B1 cells were sorted and cultured in the presence of R848 and apoptotic cells. IL-10 was measured after 72 h. WT are shown with open bars and CR2^−/−^ shown with filled bars. Data representative of *n* = 3. Statistical differences were determine by unpaired Student’s *t*-test *P < 0.05, **P < 0.01, and ***P < 0.001.

For all staining, cells were stained in PBS with 2% FCS for 20 min at 4°C. BD Aria II was used for flow sorting and BD LSRII was used to collect data. For sorted cells, debris and dead cells were excluded using FSC-SSC. Doublets were excluded using both FSC and SSC singlet gating, then CD19^+ve^ B cells isolated. For Figures 1C and 2A, CD4 and CD3 stains were also included to exclude contaminating T cells. Antibodies used were specific for and labeled with CD21-FITC, CD3-PE TxR, CD4-PE, IgM-APC, GM-CSF-PE, IL-10-PE, IL-17-alexa fluor647, IFN-γ-FITC (BD Biosciences); CD11b-BV570, CD11b-PE Cy5, CD19-PE, CD19-BV605, CD1d-PerCP Cy5.5, CD21/35-APC, CD23-PE, CD23-alexa fluor 647, CD23-PE Cy7, CD3-FITC, CD3 PE/Dazzle 594, CD38-PE, CD4-PE, CD4-BV605, CD40-PE Cy7, CD43-FITC, CD43-APC, CD5-PE Cy5, CD80-PerCP Cy5.5, CD86-BV421, TIM1-PE, TNFα-BV605 (Biolegend); CD21/35-APC efluor780, CD24-APC efluor780, CD25-efluor450, CD9-APC, DO11.10 TCR-Biotin, IgD-efluor450, MHCII-PE Cy5, CD19-eFluor450, F4/80-APC, IFN-γ-PE Cy7, streptavidin-PE (eBioscience); IgM-TxR, IgM-alexa fluor647 (Southern Biotech); IgM-alexa488, cell tracker green (Molecular Probes), and CFSE (fluka). All analysis was performed using FlowJo Software.

### IC Staining

Cells were stimulated for 4.5 h total with PMA [Sigma (20 ng/ml)] and Ionomycin [Sigma (1 μg/ml)]. After 1 h stimulation, Brefeldin A [Sigma (1 μg/ml)] was added for the remaining 3.5 h. Surface staining was performed before resuspending in fixation and permeabilization solution for 20 min (Cytofix/Cytoperm kit, BD Biosciences) followed by IC staining. All IC antibodies were used at 1:100 for 30 min in 1× Perm/Wash buffer (Cytofix/Cytoperm kit, BD Biosciences).

### Thymus AC Generation

Thymi were removed from 4- to 6-week-old syngeneic mice, teased into single cell suspensions and cultured for 18 h in IMDM (supplemented with 100 U/ml penicillin, 100 μg/ml streptomycin, 20 mM 2-mercaptoethanol, and 10% heat inactivated FCS). Cells prepared in this way give an average of 43% Annexin-V (AnV)^+ve^/propidium iodide (PI)^−ve^ ACs and <5% AnV^+ve^/PI^+ve^ secondary necrotic cells (14). Where ACs are needed for experiments involving C1q^−/−^ ACs were generated following culture in X-Vivo 15 media without serum.

### Jurkat AC Generation

Treatment with anti-CD95 (1 μg/ml) induced Jurkat cells to become apoptotic after 18 h in culture with serum free RPMI (supplemented with 2 mM l-glutamine, 100 U/ml penicillin, and 100 μg/ml streptomycin). At this point 80% were AnV^+ve^. Cells used in phagocytosis or interaction assays were labeled with cell tracker green (Molecular Probes) prior to induction of apoptosis.

### B Cell Isolation

Mouse splenocytes were “teased apart” to obtain single cell suspensions and depleted of red cells with red cell lysis buffer (Sigma-Aldrich, St. Louis, MO, USA). B cells were isolated from spleen single cell suspensions or peritoneal lavages using positive selection with CD19^+^ microbeads as per manufacturer’s instructions (Miltenyi Biotech). Cells were then further sorted by FACS as per figure legends.

### *In Vitro* TLR Stimulation Assays

1 × 10^5^ FACS sorted B cells were cultured in complete IMDM along with 1 × 10^6^ apoptotic thymocytes and the relevant stimulation. After 72 h, supernatants were removed and cytokine levels checked by ELISA (All R&D Duoset ELISA kit except IL-35 Biolegend). Cells were stimulated with: TLR7/8 ligand R848 [InVivogen (0.1 μg/ml for B cell stimulation, 0.5 μg/ml for macrophage stimulation)], the mouse TLR9 ligand CpGB [ODN1826 Eurofins MWG Operon (1 μg/ml)], LPS [Sigma (2 μg/ml)], MOG35–55 [Cambridge Research Biochemicals (20 μg/ml unless otherwise stated)], or OVA323–339 [Cambridge Research Biochemials (2 μg/ml unless otherwise stated)].

### *In Vivo* IL-10^+ve^ B Cell Antibody Generation

IL-10-GFP mice were injected IV on D0, D2, and D5 with 20 × 10^6^ apoptotic thymocytes. On D7 mice were sacrificed and blood, peritoneal lavage and spleens harvested. Splenic CD19^+ve^ B cells were FACs sorted into IL-10-GFP^+ve or −ve^ fractions, phenotyped and cultured for 10 days in the presence of MegaAPRIL [Adipogen (200 ng/ml)], CpGB [ODN1826 Eurofins MWG Operon (1 μg/ml)], and IL-4 [R&D System (50 ng/ml)], after which culture supernatants were checked for IgM and IgG levels (Ready-SET-Go ELISA kit eBioScience).

### Hybridoma Generation

Highly purified IL-10-GFP^+ve^ B1a cells, generated *in vivo*, were fused with SP2/0 cells to generate hybridomas. Cells were cultured with peritoneal macrophages for 7 days and supernatants screened (see Figures S3C,D in Supplementary Material) prior to subcloning. Colonies were selected on their ability to bind to ACs.

### Immunofluorescence

Apoptotic Jurkats were incubated with 20 μg/ml of IgM derived from the hybridoma supernatant followed by anti mouse IgM-alexa fluor 488 (1:400 dilution. Molecular probes). The plasma membrane was stained with Cell Mask Deep Red plasma membrane stain (1:1,000 in PBS) for 10 min at 37°C and following fixation with 3.7% formaldehyde the nucleus stained with DAPI. Cells, mounted in ProLong^®^ Gold Antifade Mountant were visualized on a Leica SP5 confocal microscope.

### Antigen ELISA

EIA/RIA (costar) 96-well plates were coated with 2 μg/ml (in 50 μl PBS) antigen overnight then blocked (PBS + 1% BSA) for 1.5 h. Antigens used were malondialdehyde-modified low-density lipoprotein (MDA-LDL), oxidized-LDL (Ox-LDL), citrullinated fibrinogen (Cambridge Biosciences); DNA, ssDNA, thyroglobulin, yeast RNA, alpha Actinin (Sigma); La, Ro, Smith, histone (Arotec Diagnostics); rheumatoid factor (Thermofisher Scientific), CWPS (Oxford Biosystems), ApoH (R&D Systems), PC-BSA (2B Scientific), and AnV (eBiosciences). Serum samples were initially diluted at 1:250. For *in vitro*-generated antibodies, supernatants were initially diluted 1:2. For hybridoma-generated antibodies, IgM was determined and supernatants were diluted to 0.5 μg/ml prior to serial dilutions. Secondary antibody (1:1,000 dilution) were either anti mouse IgM-HRP (Southern Biotech) or anti mouse IgG-HRP (Zymed). MRL/lpr mouse serum (diluted 1:250) was used as a positive control while IgM derived from IL-10^–ve^ clones that did not bind to ACs were used as negative controls.

### *In Vivo* Antigen Challenge Experiments

Single cell suspensions of lymph node cells (from OVA Tg mice) were prepared and 5 × 10^6^ cells injected along with 20 × 10^6^ ACs (IV). For experiments with C1q^−/−^ mice, C1q^−/−^ thymocytes were cultured in serum free X-Vivo 15 media. Mice were immunised with 50 μl OVA323–339 peptide (1 mg/ml) emulsified in an equal volume of complete Freund’s adjuvant (CFA) sc into each hind leg. On D7 splenic single cell suspensions were generated prior to restimulation using 1 × 10^6^ splenic cells along with OVA323–339 peptide at 4, 2, 1, 0.5, and 0 μg/ml. After 72 h, cytokines were measured (R&D duoset ELISA).

### T Cell Proliferation

Splenic cells were labeled with 1 μM CFSE before setting up in culture with 2 μg/ml OVA_323–339_. Cells were harvested every 24 h and further stained with CD3 and Ly5.1 (for OTII). The mean florescence intensity (MFI) of CFSE for CD3^+ve^ Ly5.1^+ve^ cells was measured using flow cytometry.

### B Cell Interaction Assays

Purified B cells were incubated with cell tracker green labeled AC-Jurkats in a ratio of 1:5. After 4 or 24 h, cells were stained for CD19 and CD43. Interaction was measured using flow cytometry by determining the percentage of B cells which were also positive for cell tracker green, indicating interaction between the B cell and AC.

### Preparation of Primary Bone Marrow-Derived Macrophages (BMDMs)

Bone marrow-derived macrophages were isolated from the hind leg bones of C57BL/6 mice and cells cultured in compete IMDM + 10% L929-conditioned media (containing M-CSF) for 7 days in flat bottom plates.

### Phagocytosis Assay

1 × 10^6^ cell tracker green labeled AC-Jurkats were incubated with 20 μg/ml of hybridoma derived IgM and cocultured with BMDM for 30 m.

### Macrophage-B Cell Cocultures

Peritoneal CD43^+ve^B1a or splenic follicular B (FOB) cells were activated with R848 (0.1 μg/ml) for 24 h and then cocultured with R848 (0.5 μg/ml) activated BMDM for 18 h. 20 μg/ml anti-IL-10 or isotype control antibody (Biolegend) was added for length of culture. Supernatants were assessed for released cytokines by ELISA (R&D duoset) and cells were stained for IC cytokines.

### Induction and Assessment of EAE

Experimental autoimmune encephalomyelitis was induced as described previously (15). On D3/4 mice were either given 1 × 10^5^ CD43^+ve^ B1 cells or splenic FOB, that had been previously activated with R848 (0.1 μg/ml) for 48 h. Following sacrifice, harvested organs were weighed and single cell suspensions from the spleen and LN generated. Spinal cords were collagenase/DNase digested before running over 70/30% Percoll gradient to obtain leukocytes. Re-stimulation assays were set up using 1 × 10^6^ splenic or LN cells or 1 × 10^5^ spinal cord cells along with MOG35–55 peptide at 20, 10, 5, 2.5, and 0 μg/ml. After 72 h, cytokines were measured (R&D duoset ELISA) and IC staining performed.

### Bhlhe41 mRNA Determination

RNA was extracted from 10^5^ purified cells [TRI^®^ Reagent (Ambion)] followed by reverse transcription [High Capacity cDNA reverse transcription kit (Applied Biosystems)]. Bhlhe41 cDNA levels were quantitated by Taqman^®^ Gene Expression Assay predesigned primers (Mm00470512_m1) with intra-sample expression normalized to Eukaryotic 18S rRNA Endogenous control (FAM™/MGB probe) and run on an Applied Biosystems 7900HT Fast-Real Time System using SDS software (v2.4). Data were analyzed using the comparative CT method (Δ/ΔCT), where fold differences in gene expression between FOB and other B cell populations (ΔCT) were normalized to CT values of the 18S rRNA reference gene.

### Statistical Analysis

Experimental repeats are given under each experiment and ranged from 3 to 12. Data are expressed as mean and SEM. Statistical significance between the groups was assessed by GraphPad Prism Version 7.0 using the appropriate analysis as stated in the figure legends. *P*-values: *0.01–0.05, **0.001–0.01, and ***<0.001.

## Results

### ACBregs Are Primarily B1a Cells

To identify naturally occurring populations of ACBregs, we sorted splenic B cells into transitional 2 marginal zone precursor B cells (T2-MZP-CD21^hi^CD24^hi^CD23^hi^), FOB cells (CD21^lo^CD24^lo^CD23^+ve^), and B cells found within the marginal zone (CD21^hi^CD24^hi^CD23^lo^) (Figure 1A, i; Figure S1A in Supplementary Material). Subsequent stimulation of these B cell subsets with the TLR7/8 ligand R848 or the TLR9 ligand CpGB in the presence of ACs significantly augmented the level of IL-10 secretion from CD21^hi^CD24^hi^CD23^lo^ splenic B cells (Figure 1A, ii–iii, population A3), but barely at all from either T2-MZP B (Figure 1A, ii–iii, population A4) or FOB cells (Figure 1A, ii–iii, population A1). To further define the surface markers expressed by ACBregs, highly pure populations of splenic B cells were also sorted according to their expression of CD21, IgM, and IgD (Figure 1B, i; Figure S1B in Supplementary Material). ACs induced the highest secretion of IL-10 (but not IL-35), from activated CD21^hi^IgM^hi^IgD^lo^ B cells (Figure 1B, ii–iii, population C4).

To identify *in vivo*-derived ACBregs, apoptotic thymocytes were administered intravenously to mice and splenic IL-10^+ve^ and IL-10^−ve^ B cells harvested 7 days later (Figure S1C in Supplementary Material for sort strategy and purity). At this point the percentage of IL-10^+ve^ B cells had doubled (Figure 1C, i). Further analysis of the surface markers indicated that approximately 80% of the splenic IL-10^+ve^ B cells were B1a cells (CD19^hi^CD43^+ve^CD5^+ve^CD23^low^CD38^+ve^CD25^+ve^CD1d^+ve^IgD^low^IgM^hi^) (Figure 1C, ii; red line and Figure S1Ciii–iv in Supplementary Material). In line with possible *in vivo* activation (see Figure S1Cv in Supplementary Material), CD21 expression was reduced following AC infusion (30). Compared to IL-10^−ve^ B cells, ACBregs also expressed more CD86, Tim1, CD9, CD80, and CD40 (Figure 1C, iii).

To assess the differential responses of activated splenic B cells to ACs, highly purified splenic MZB (CD19^+ve^CD43^−ve^IgD^low^IgM^hi^), B1a (CD19^+ve^CD43^+ve^IgD^low^IgM^hi^), or FOB (CD19^+ve^CD43^−ve^IgD^+ve^IgM^lo^) B cells were cocultured with OVA peptide, OVA-specific T cells, with and without ACs. Cultures containing B1a cells generated significantly more IL-10 than MZB (Figure 1D). However, MZB were also able to respond to ACs and secreted significantly more IL-10 than those cultures containing FOBs. This indicates that while splenic B1a B cells make up a major component of ACBregs, activated splenic CD43^−ve^ MZB cells can also respond to ACs by secreting IL-10.

Similar results were seen in the peritoneal cavity (PerC). Following an injection of ACs the percentage of IL-10^+ve^ B cells in the PerC had increased by 50% at day 7 when compared to naive mice (Figure 1E, i). Approximately 96% of the IL-10^+ve^ B cells were CD43^+ve^ and had the markers of PerC B1a B cells (Figure 1E, ii; red line and Figure S1Di–iii in Supplementary Material). Again, the expression of CD86, Tim1, CD9, CD80, MHCII, and CD40 were also increased compared to IL-10^−ve^ B cells (Figure 1E, iii). As expected, the CD5 staining on the B1a B cells was lower than that found on T cells (Figure S1Ei in Supplementary Material). However, prior treatment with ACs did not alter the percentage of cells that expressed CD43 or CD5 (Figure S1Eii–iii in Supplementary Material). In keeping with a recent report (31), CD43^+ve^ splenic and peritoneal B1a B cells preferentially expressed the transcription factor Bhlhe41, when compared to splenic FOB or marginal zone B cells and peritoneal CD43^−ve^ B cells (Figure 1F). *In vitro*, the percentage of IL-10^+ve^ B1a B cells is augmented by apoptotic thymocytes, but this requires a second signal such as TLR7/8 activation (Figure 1G).

### B1a B Cells Augment T Cell IL-10 Production

We next asked if B1a B cells, isolated following an *in vivo* inflammatory immune response, could still induce antigen-specific T cells to secrete IL-10. Ovalbumin peptide (OVA_323–339_)-specific D011.10 T cells were transferred into mice that had been given an intravenous injection of ACs at the time of OVA_323–339_ peptide (emulsified in CFA) immunization. A week later, PerC B cells were sorted into CD19^hi^CD43^+ve^ and CD19^+ve^CD43^−ve^ subsets (Figure S2A in Supplementary Material). Splenic CD19^+ve^ B cells were harvested and sorted into naive CD19^+ve^IgD^hi^ B cells and CD19^hi^CD43^+ve^ splenic B1a cells (Figure S2B in Supplementary Material). These B cell subsets were used as antigen-presenting cells (APCs) to stimulate naive OVA_323–339_-specific T cells and IL-10 production was assessed after 72 h (Figures 2A,B, i,iii). IC staining confirmed that the highest percentage of IL-10 producing cells was still seen among the splenic and PerC CD43^+ve^ B1a B cells. These same B1a B cells also induced a significantly higher percentage of CD4^+ve^ T cells to produce IL-10 (Figures 2A,B, ii). Hence splenic or PerC ACBregs were able to induce antigen-specific T cells to secrete IL-10, even following a proinflammatory *in vivo* stimulus.

### ACBregs Do Not Require Tim1 But Are Augmented in C1q^−/−^ and CR2^−/−^ Mice

T cell Ig and mucin domain (Tim1) has been reported to identify ACBregs and the loss of this molecule has been associated with impaired IL-10 production and the promotion of inflammatory T cell responses (32, 33). To ask if splenic B cells required Tim1 expression to induce IL-10 secretion following interaction with ACs, IgM^hi^IgD^lo^CD21^hi^ splenic B cells from WT and Tim1-deficient (Tim1^−/−^) mice were stimulated with the TLR7/8 ligand R848 or used as APCs to stimulate naive OVA_323–339_-specific T cells *in vitro*. We did not detect any difference in the ability of TLR or T cell activated Tim1^−/−^ B cells to respond to ACs by secreting IL-10 (Figure 2C, i–ii) [on both the BALB/c or C57BL/6 background (see Figure S2Di–iv in Supplementary Material)]. Nor was there a difference in regulatory cell surface markers or their ability to interact with ACs (Figures S2E,F in Supplementary Material). Similar results were seen when splenic B cells were stimulated with the TLR9 ligand CpGB or the TLR4 ligand LPS (Figure S2Gi–ii in Supplementary Material). When ACs were given *in vivo* at the time of OVA_323–339_/CFA immunization (as described for Figure 2A) no differences in IL-10 secretion (Figure S2H in Supplementary Material) or T cell proliferation upon re-stimulation (Figure S2I in Supplementary Material) was seen; leading us to conclude that Tim1 is not required for innate-like regulatory B cells to induce regulation.

Mammalian DNA has been shown to bind to CR2/CD21, which is most highly expressed by MZB and PerC B1 B cells (34, 35). ACs express DNA containing chromatin complexes on their cell surface and altered expression of CR2 is associated with SLE in mice (36). In addition, complement proteins, including C1q, bind to late ACs and increase their uptake by phagocytes (21, 37, 38); the loss of which is believed to contribute to the increased risk of SLE (20). Hence complement may also play an essential role in regulatory B cell function. To test this, we assessed the cytokine responses of WT or CR2^−/−^ splenic B cells, activated with TLR7/8 or TLR9 and cocultured with ACs. To avoid bias that may occur with B cell surface markers from these knockout mice, we assessed CD19^+^IgD^lo^IgM^hi^ B cells. In contrast to TIM1^−/−^ B cells, TLR stimulated CD19^+^IgD^lo^IgM^hi^ CR2^−/−^ B cells secreted significantly more IL-10, both with or without added ACs (Figure 2D; Figure S2K in Supplementary Material). Similar results were seen following the *in vivo* immunization with OVA_323–339_ peptide in CFA, OVA_323–339_-specific T cells and an infusion of ACs. Re-stimulation of splenocytes from these immunized mice a week later also generated significantly more IL-10 than WT controls (Figure 2E). The increase in IL-10 secretion may have resulted from the significantly higher percentage of splenic CD43^+ve^ B1a cells found among the CD19^+^IgD^lo^IgM^hi^ B cells (Figure 2F, i). The percentage of peritoneal B1 cells was also significantly increased (Figure 2F, ii). When equivalent numbers of splenic (CD19^+^IgD^lo^IgM^hi^CD43^+^) or PerC (CD19^hi^CD43^+^) B1 cells were isolated from CR2^−/−^ mice or WT controls and activated with TLR7/8 (R848) and ACs, similar amounts of IL-10 were generated (Figure 2G). Comparable results were seen with splenic CD21^hi^IgD^lo^IgM^hi^ B cells from C1q^−/−^ mice (Figures S2L–N in Supplementary Material). This indicates that while ACBreg function is not affected by the absence of TIM1, it is augmented in both C1q- and CR2-deficient mice, likely as a result of the higher percentage of splenic and peritoneal B1a cells, able to respond to ACs.

### Regulatory B1a Cells Are Specific for AC Expressed Neoantigens

**Figure 3:**
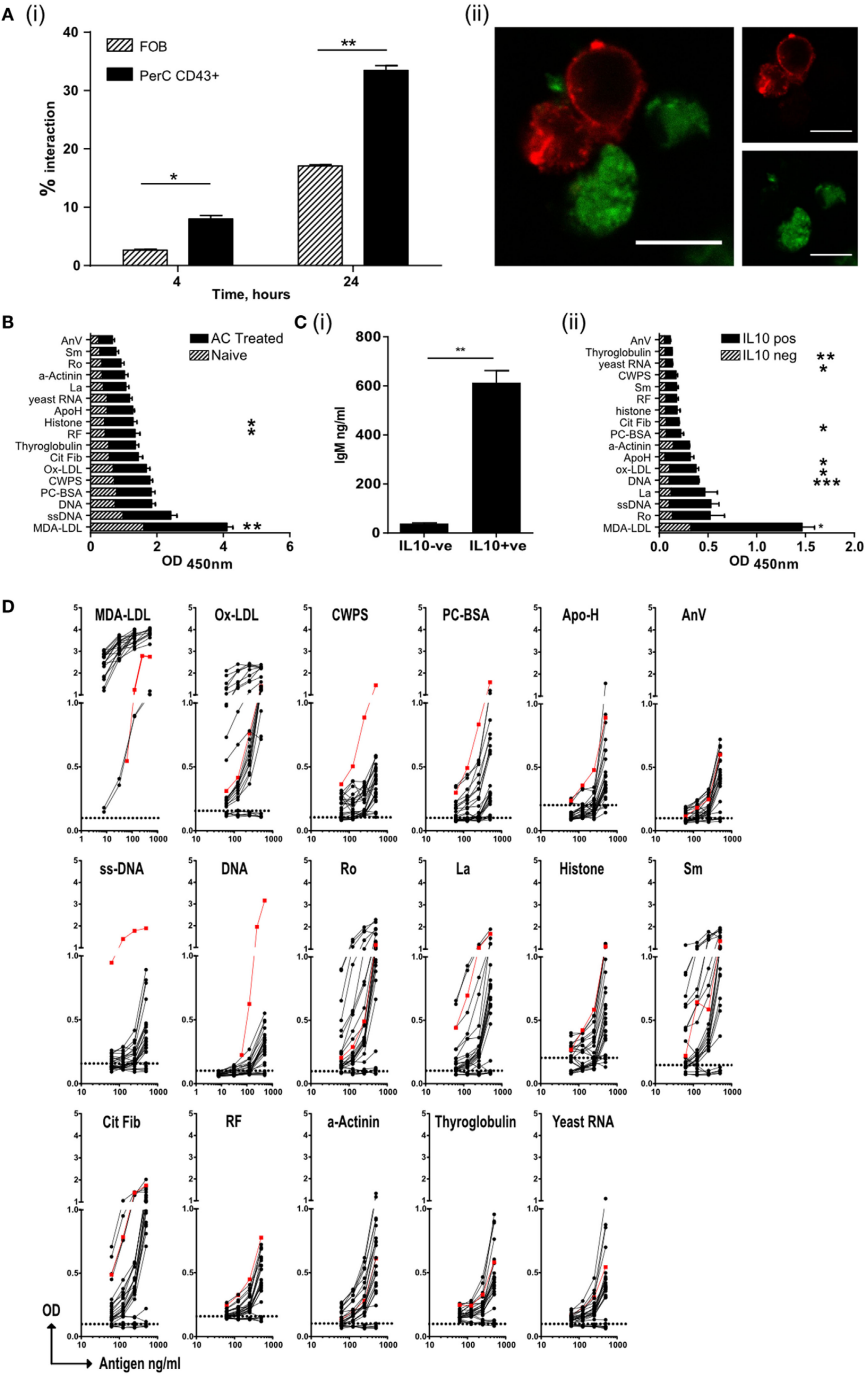
Regulatory B cells that recognize apoptotic cells, produce both IL-10 and IgM autoantibodies. **(A)** Splenic follicular B (FOB) cell (patterned bars), or peritoneal cavity B1a cells (solid bars), activated with R848, were cultured with CM-green labeled apoptotic cell (AC) Jurkat cells. (i) B cell–AC interaction was assessed by flow cytometry at 4 and 24 h. Representative plots can be found in Figure S3Ai in Supplementary Material. (ii) Cells were also visualized by confocal microscopy to show B/AC interaction. IgM^+ve^ B cell (red), AC (green). Scale bar represents 20 μm. Single color controls can be found in Figure S3Aii in Supplementary Material. Data representative of two separate experiments. **(B)** ELISA of serum IgM from mice given AC infusions 7 days earlier. Naive untreated mice are shown in the patterned bars and AC-treated mice in the solid bars. Data pooled of five individual mice and from two experiments. **(C)** IL-10^+ve^ and IL-10^−ve^ CD19^+ve^ B cells were isolated from spleens on D7 post-AC injection and cultured with MegaAPRIL, IL-4, and CpG. (i) Supernatants were tested for IgM on Day 10. Data are representative of *n* = 3. (ii) ELISA of secreted IgM from **(C)** (i). IgM from IL-10^−ve^ cells is shown with patterned bars and IL10^+ve^ cells with solid bars. Data representative of *n* = 3 and pooled from two individual experiments. **(D)** CD19^+ve^ IL-10^+ve^ B cells were fused with SP0/2 cell line to produce hybridomas as illustrated in Figure S3C in Supplementary Material. Clones, which produced antibody that bound apoptotic cells, were further sub-cloned and screened again for apoptotic cell binding (Figure S3D in Supplementary Material). Positive binding clones were then checked for IgM to specified autoantigens using an equal concentration of IgM per clone tested. Binding from IL-10^−ve^ B cell clones were used as a negative control (shown with dotted line on each graph). No IgG was detected (data now shown). Positive control (red line) serum from MRL-lpr/lpr lupus mouse was included. Data are pooled from three individual experiments.

Regulatory B cell function depends on the activation of endosomally located TLRs (15). Peritoneal B1 B cells have been reported to internalize beads and bacteria (39), but we could not detect whole ACs or apoptotic bodies within them (data not shown). However, in comparison to follicular cells, peritoneal B1a B cells made prolonged contact with ACs at both 4 and 24 h following coculture; with significantly more B1a cells still firmly bound to ACs (Figure 3A, i–ii; Figure S3A in Supplementary Material). These stable interactions may allow the recognition of AC expressed neoantigens *via* the B cell receptor (40), which we and others have previously reported to be required for regulatory B cell function (14, 41, 42).

Apoptotic cells express a range of self-antigens on their cell surface, including DNA, the proteins Ro and La, and the phospholipid phosphatidylserine, all of which are potent autoantigens in SLE and Sjogren’s syndrome (17, 19). Following PCD membrane LDLs also undergo peroxidation, resulting in the formation of neoepitopes including MDA-LDL, Ox-LDL, and phosphorylcholine (PC) (22). In contrast to the PerC, the spleen is a major site of antibody production by B1a B cells (28) and following an infusion of ACs, a rise in the titer of IgM (but not IgG) with specificities typical of NAbs was observed in the serum (Figure 3B) (22). To ask whether IL-10^+ve^ splenic B cells were the source of these antibodies, IL-10^+ve^ and IL-10^−ve^ splenic B cells were sorted seven days after the administration of ACs (as shown in Figure S1Ci–ii in Supplementary Material). These cells were cultured *in vitro* with a proliferation inducing ligand (APRIL), IL-4, and CpGB to stimulate antibody secretion. After 10 days, supernatants were harvested and tested for the specificity of secreted immunoglobulin. Only IL-10^+ve^ splenic B cells secreted significant quantities of IgM (Figure 3C, i) but not IgG (data not shown). The antibodies secreted into the supernatants were of similar specificities to those antibodies seen in the serum of mice injected with ACs (Figure 3C, ii); confirming that IL-10^+ve^ regulatory B cells respond to AC expressed neoantigens by secreting NAbs. To further assess the specificity of these regulatory cells, IL-10^+ve^ and IL-10^−ve^ splenic B cells were sorted from mice that had been given an infusion of ACs 7 days earlier. As noted before, IL-10^+ve^ B cells had the markers of B1a B cells (Figure S3B in Supplementary Material). B cells were then fused with the SP2/0 cell line to form hybridomas (as illustrated in Figures S3C,D in Supplementary Material). Individual clones were tested for reactivity to a variety of epitopes found on ACs and within the NAb repertoire. Again, only IgM secreted by the hybridomas originating from the IL-10^+ve^ (but not IL-10^−ve^) splenic B1a cells, showed cross-reactivity with a range of epitopes expressed on ACs, particularly MDA-LDL, Ox-LDL, and the autoantigens Ro, La, histones, and Smith (Sm) protein (Figure 3D). Hence, ACBregs that respond to ACs are B1a cells that secrete both IL-10 and IgM with specificities that resemble NAbs.

### ACBregs Inhibit Macrophage Proinflammatory Function

**Figure 4:**
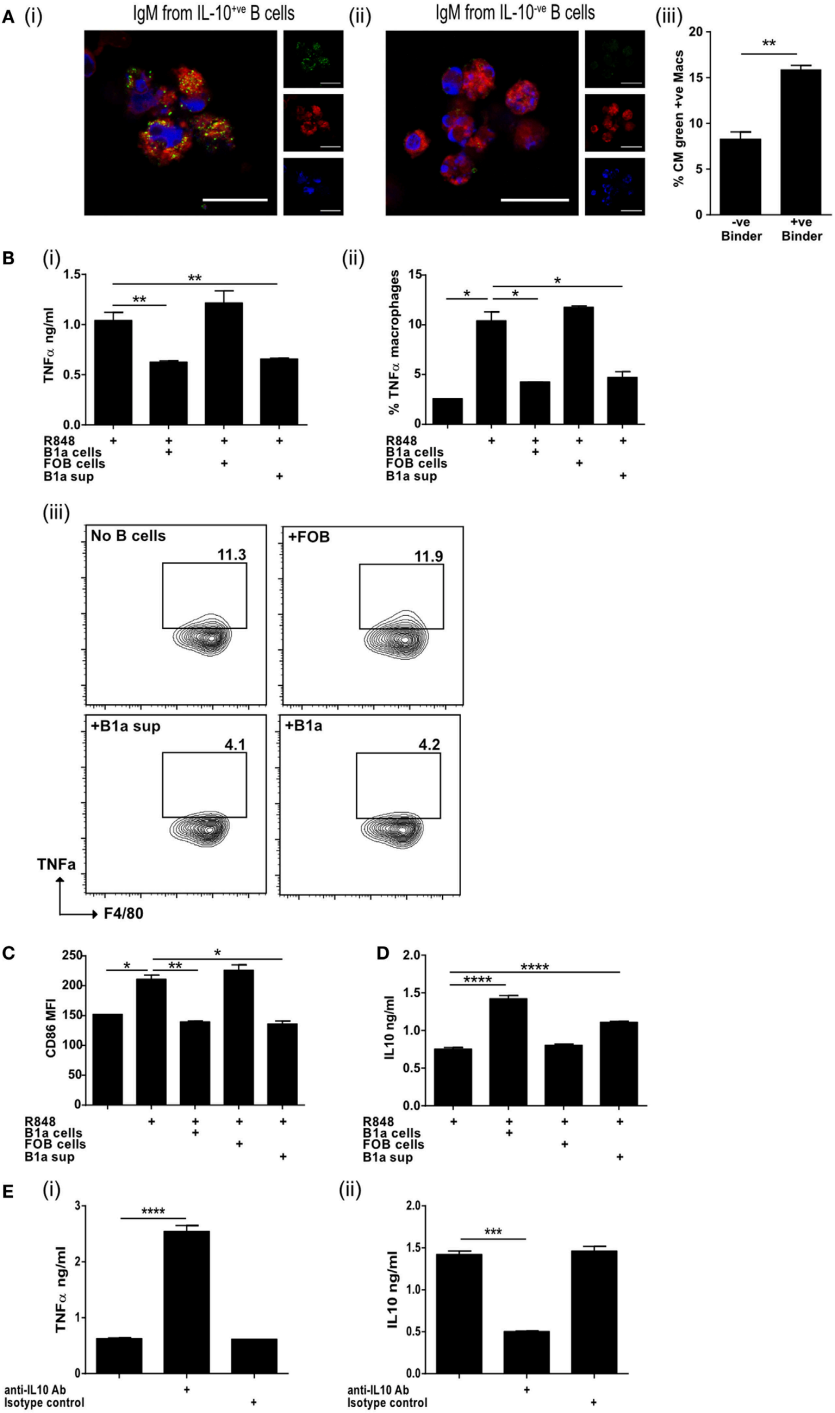
IL-10 from regulatory B cells can inhibit macrophage activation. **(A)** Supernatants from IL-10^+ve^ and IL-10^−ve^ B cell hybridomas generated in Figure 3D were pooled and binding to apoptotic cells tested by microscopy (i and ii). Cell membrane is stained with cell mask plasma membrane dye shown in red, IgM green and DAPI blue. Scale bar represents 20 μm. Single color controls can be found in Figure S4 in Supplementary Material. Supernatant was also used in macrophage phagocytosis assays (iii). Data representative of *n* = 3. **(B)** Splenic follicular B (FOB) cells (CD19^+ve^CD23^+ve^CD21^lo^CD43^−ve^) or peritoneal cavity B1a cells (CD19^+ve^CD43^+ve^CD5^+ve^) were FACs sorted and activated with R848 for 24 h, prior to coculture for a further 18 h with R848 activated bone marrow-derived macrophages (BMDM). Supernatants obtained from cultures of R848 activated B1a cells (B1a sup) was also added to R848 stimulated BMDM. TNFα was measured in the supernatants of cocultures (i) and by intracellular staining of F4/80^+ve^ CD19^−ve^ BMDM at end of culture (ii and iii). **(C)** Surface expression of CD86 (MFI) on F4/80^+ve^ BMDM at end of culture. **(D)** IL-10 was measured in the supernatants of cocultures. **(E)** TNFα (i) and IL-10 (ii) was measured in the supernatants of cocultures (BMDM and B1a cells) in the presence of anti-IL-10 antibody or isotype control. Data representative of two experiments with duplicate treatments per experiment except **(E)** where *n* = 3. Statistical differences were determined by unpaired Student’s *t*-test *P < 0.05, **P < 0.01, and ***P < 0.001.

Natural antibodies augment the clearance of ACs (43) and IgM secreted by ACBregs preferentially bound to ACs, while the antibodies derived from IL-10^−ve^ splenic B cells did not (Figure 4A, i–ii; Figures S4A,B in Supplementary Material). The phagocytosis of ACs by BMDM, that had bound ACBreg derived IgM, was also significantly increased (Figure 4A, iii), as expected (44). BMDMs are particularly sensitive to the effects of IL-10, which is largely responsible for the anti-inflammatory response, acting *via* the IL-10 receptor and increasing signaling through the JAK1/STAT3 cascade (45). In contrast, TLR7/8 stimulation with R848 induces BMDMs to secrete TNFα. We noted that, in distinction to FOB cells, the coculture of B1a B cells with BMDMs, where both cell types had been activated with R848, resulted in a significant decrease in the amount of TNFα secreted into the culture medium (Figure 4B, i), as well as the percentage of macrophages (F4/80^+^CD19^−^) positive for IC TNFα (Figure 4B, ii–iii). Supernatants transferred from R848 activated B1a B cells to similarly activated macrophages also diminished TNFα production (Figure 4B, i–iii), as well as the activation marker CD86 (Figure 4C). IL-10 secreted by activated B1a cells (Figure 4D) was required to prevent TNFα secretion, as evidenced by the loss of macrophage TNFα suppression in the presence of anti-IL-10 antibodies (Figure 4E, i–ii).

### Activated B1a B Cells Suppress Autoimmunity

**Figure 5:**
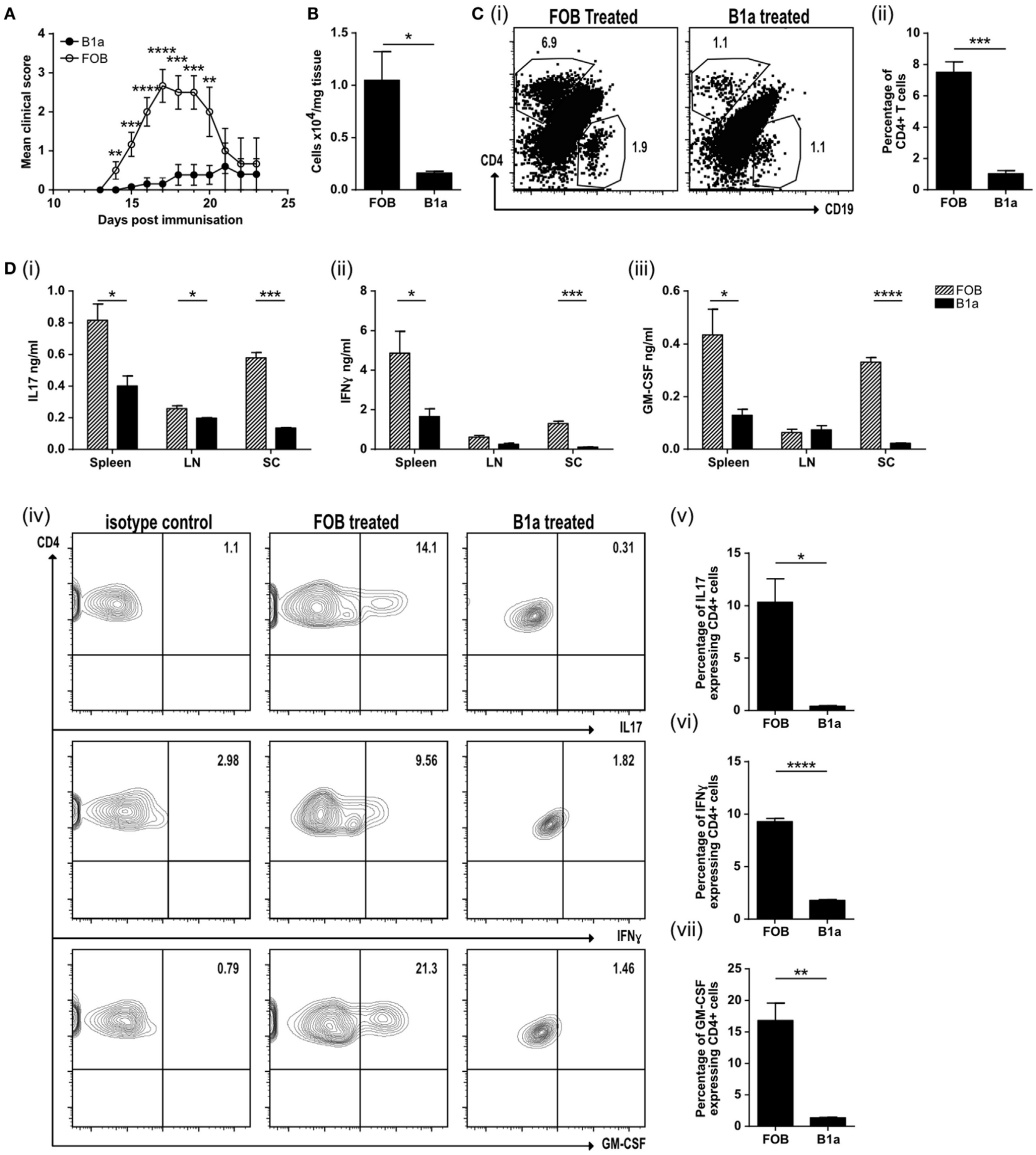
B1a cells protect mice from experimental autoimmune encephalomyelitis (EAE) by reducing cell inflammatory cell influx into the spinal cord. **(A)** Mean clinical score of mice treated with either splenic follicular B (FOB) cells (CD19^+ve^CD23^+ve^CD21^lo^CD43^−ve^) (open circles) or peritoneal cavity B1a cells (CD19^+ve^CD43^+ve^CD5^+ve^) (filled circles) that had been treated with the TLR7/8 ligand R848 for 48 h *in vitro*. Data representative of six individual mice. **(B)** Number of cells per mg of spinal cord tissue. **(C)** CD4^+ve^ T cells and CD19^+ve^ B cells within the spinal cord. Representative plots can be found in (i) and pooled data from three individual mice graphed (ii). **(D)** Cells were restimulated with 20 μg/ml MOG for 72 h before measuring, (i) IL-17, (ii) IFN-γ, and (iii) GM-CSF in the supernatants. Data from FOB-treated mice is shown with patterned bars, and B1a-treated mice shown with solid bars. Cultures were also IC stained for cytokines. Representative plots from spinal cord CD4^+ve^ cells are shown in (iv) and data pooled from three individual mice graphed for IL-17 (v), IFN-γ (vi), and GMCSF (vii). Statistical differences were determine by unpaired Student’s *t*-test *P < 0.05, **P < 0.01, and ***P < 0.001.

The neuroinflammatory disease model EAE is driven by both activated, antigen-specific T cells and macrophages. We next asked if activated B1a could limit auto-immune mediated inflammation. Mice were immunized with MOG peptide emulsified in CFA (MOG/CFA) to induce EAE. PerC B1a cells and splenic FOB cells were stimulated with the TLR7/8 ligand R848 for 48 h *in vitro* and either 10^5^ B1a cells or 10^5^ FOB cells were injected intravenously 3–4 days following immunization with MOG/CFA. Despite activating the B1a cells with a proinflammatory stimulus (*via* TLR7/8 with R848), clinical disease severity was significantly reduced (Figure 5A). On day 20 the spinal cords of control FOB cell-treated mice (FOB) also contained a greater number of total cells compared to those given B1a cells (Figure 5B), though cell numbers in the spleen and draining lymph nodes were unchanged (data not shown). The spinal cords were further analyzed for CD4^+ve^ and CD19^+ve^ cells. Compared to B1a-treated mice, the percentage of CD4^+ve^ T cells in the spinal cords of mice given control FOB cells was more than six times higher, while CD19^+ve^ B cell infiltration was approximately halved in B1a-treated mice (Figure 5C, i–ii). Lymphocytes from the spleen, draining lymph nodes and spinal cord were re-stimulated with MOG peptide for 72 h and the cytokines IL-17, IFN-γ, and GM-CSF quantified by ELISA. In keeping with the reduced inflammation seen clinically, B1a-treated mice generated significantly less proinflammatory cytokines than control FOB cell-treated mice in all organs tested, but particularly the spinal cords (Figure 5D, i–iii). IC cytokine staining confirmed that a source of these cytokines was the CD4^+ve^ T cell, which again showed a significantly lower production of IL-17, IFN-γ, and GM-CSF in B1a-treated mice (Figure 5D, iv–vii). This confirms that activated B1a B cells are able to suppress autoimmune mediated inflammation when administered after the initiation of EAE.

## Discussion

This report pinpoints self-reactive, CD43^+ve^ B1a cells, which express the transcription factor Bhlhe41, found in the spleen and PerC, as a major responder to ACs. Of the remaining 20% of splenic CD43^−ve^ B cells, that similarly responded to ACs, it is likely that many of these reside within the MZB cell population. Dying cells are a potent source of expressed neoantigens (17, 46), which are recognized by ACBregs *via* their BCR and TLR receptors, leading to the secretion of both IL-10 and NAbs (14, 15). We show that this both accelerates the clearance of ACs by macrophages and simultaneously reduces macrophage proinflammatory responses. Furthermore, ACBregs induce antigen-specific T cells to secrete IL-10 and suppress the induction of autoimmune CNS inflammation. Hence AC-specific B1a B cells fulfill two important homeostatic functions; they ensure, *via* Nab secretion, the rapid disposal of apoptotic corpses and, *via* IL-10 secretion, they prevent inflammatory autoimmune responses. Activated macrophages orchestrate much of the tissue injury seen in autoimmune diseases including rheumatoid arthritis, multiple sclerosis, psoriatic arthritis and inflammatory bowel disease (47). IL-10 is a key regulator of activated macrophages (48) and as such, activated IL-10 secreting ACBregs are likely to play a physiologically relevant role, both in preventing a break in self-tolerance and containing ongoing autoimmune responses.

In contrast, we found that T2 marginal zone precursor B cells (T2-MZB) secreted much less IL-10 in response to activation by TLR ligands and coculture with ACs. This confirms that while T2-MZB precursor B cells regulate particular immune responses, they do not play a clear role in mediating tolerogenic responses to apoptotic self (11, 49). Tedder et al. have previously described a population of IL-10 secreting “B10” cells, which are enriched in the expression of CD1d and CD5 (8, 9). They are functionally defined in mice and humans by their ability to secrete IL-10 following 5 h of *ex vivo* stimulation with lipopolysaccharide (LPS), phorbol ester, and ionomycin (PMA/Iono). The term B10 usefully includes all B cells that have the capacity to secrete IL-10 following this stimulus. However, it necessarily encompasses B cell subtypes with differing origins and functional attributes and does not enable one to determine the particular characteristics of regulatory B cells that are concerned with self-tolerance. In addition, TLR activated B cells increase their expression of CD1d (see Figure S1Cvi in Supplementary Material), which further complicates drawing firm conclusions about subset origin.

Early studies confirmed that B1a B cells were self-reactive as well as being a major source of IL-10 (3, 24, 25, 28). They constitutively express STAT3 which binds to the IL-10 promoter, further enhancing the generation of IL-10 (26, 50). Once activated, they migrate away from the coelomic cavities into the spleen, bone marrow, and other lymphoid organs, where they secrete immune modulatory IL-10 and NAbs (51, 52); so maintaining a tolerogenic environment toward AC expressed neoantigens. B1a development and NAb secretion does not require foreign antigenic stimulation, which occurs under gnotobiotic conditions (53). Indeed, it has long been suspected that ACs play an important role in their development and these data further support a central relationship between dying cells and B1a cell function.

Apoptotic cell responsive regulatory B cells generate polyclonal IgM that binds particularly well to AC expressed neoantigens including MDA-LDL, Ox-LDL, PC, Ro, La, histones, and Smith antigen; which accelerates their clearance. The prototypic NAb T15, identified over 40 years ago, arises within a week of birth in the absence of pathogens, and also binds to PC that is expressed on pneumococci (54, 55). T15-NAbs similarly bind to oxidatively modified LDL, reducing the inflammation associated with atherosclerosis (56) and arthritis (57, 58) *via* a direct effect on dendritic cells and macrophage function.

Phosphatidylserine which is exposed on the surface of ACs, is recognized by members of the TIM domain family (that includes TIM1, TIM3, and TIM4) (59). Considering previous reports of a requirement for TIM1 expression by regulatory B cells (32, 33, 60), we were surprised to find that TIM1-deficient B cells on both the BALB/c and the C57BL/6 background responded normally to ACs, both *in vitro* and *in vivo*, by secreting IL-10. These differences could have arisen from alternative gene knockout strategies, because in TIM1-deficient mice where immune regulation is altered, only the mucin domain of TIM1 has been deleted. In contrast, we analyzed TIM-1-deficient mice that lacked the full-length molecule. Complement deficiency is also believed to influence the predisposition to autoimmunity because of an inability to coat ACs with C1q or the later complement components. Such opsonization is required for the efficient clearance of ACs by phagocytes (61). Again, we were intrigued to find that, rather than a loss of regulatory B cell function, the absence of either C1q or CR1/CR2 led to enhanced IL-10 secretion. This was explained by the increased frequency of splenic and PerC B1a B cells in both knockout phenotypes and confirms that C1q or CR1/CR2 are not needed for B1a B cells to secrete IL-10 in response to ACs or TLR ligand stimulation. In relation to autoimmunity, B1a B cells in the NZB/W F1 lupus model class switch to IgG and accumulate in the spleen and target organs (62). In addition, B1a B cells are crucial in the NOD mouse model of diabetes (63). We would speculate that tissue-specific signals and/or unique pathogen derived signals combine to determine whether the response of B1a cells is predominantly regulatory (*via* IL-10/NAbs) or proinflammatory (*via* GM-CSF/NAbs) (27, 29, 64).

In summary then, we have delineated that a major population of regulatory B cells that responds to ACs are B1a cells. This population of B1a cells functions to optimize the disposal of ACs (*via* Nabs), while inhibiting macrophage proinflammatory responses and promoting regulatory T cell responses to self-antigens (*via* IL-10). If an equivalent population of B cells could be harnessed in humans, it would provide a valuable means to regulate autoimmunity and transplant rejection.

## Ethics Statement

All experiments were covered by a Project License granted by the Home Office under the Animal (Scientific Procedures) Act 1986. Locally, this license was approved by the University of Edinburgh Ethical Review Committee.

## Author Contributions

KM, JS, and SB carried out experiments. GC contributed to data analysis. DG designed the hybridoma experiment and reviewed the manuscript. MG designed the experiments, analyzed the data, and wrote the manuscript.

## Conflict of Interest Statement

The authors declare that the research was conducted in the absence of any commercial or financial relationships that could be construed as a potential conflict of interest.

## Supplementary Material

Figure S1 **(A)** Purity checks of populations A1, A3, and A4 used in Figure 1A. **(B)** Purity checks of populations C1, C3, and C4 used in Figure 1B. **(C)** Sorting strategy (i) and purity checks (ii) of splenic IL-10^+ve^ cells which were further analyzed in Figure 1C. (iii) Percentage of IL-10^+ve^ splenic CD19^+ve^ B cells which express CD43 (as a marker of splenic B1a) is 77.8%. (iv) The average mean florescence intensity (MFI) data represented in Figure 1C (ii) are shown along with the mean fold difference. (v) Change in CD21 expression postculture is shown as a histogram with unstimulated marginal zone B cells shown in black and the same cells shown after 72 h R848 culture in red. (vi) Change in CD1d expression postculture is shown as a histogram with unstimulated CD19^+ve^ cells shown in black and the same cells shown after 72 h R848 culture in red. Isotype control is shown in shaded gray. **(D)** (i) Percentage of IL-10^+ve^ peritoneal cavity (PerC) CD19^+ve^ B cells which express CD43 is 96%. (ii) The average MFI data represented in Figure 1E, ii, are shown along with the mean fold difference. (iii) Representative dot plot of peritoneal CD19^+ve^ B cell used in Figure 1E, ii, showing expression of CD5 and IL-10. **(E)** (i) Level of expression of CD5 in peritoneal CD5^−ve^ B cells (black), CD5^+ve^ B cells (red) and T cells (blue). Percentage of CD43^+ve^ (ii) or CD5^+ve^ (iii) PerC B cells in Naiïve or apoptotic cellAC-treated mice used in Figure 1E and (S1D).

Figure S2 **(A)** Purity checks of peritoneal cavity (PerC) CD43^−ve^ and CD43^+ve^ (i) and CD19 expression of sorted populations with CD43^−ve^ in black and CD43^+ve^ in red (ii) used in Figure 2A. **(B)** Gating strategy of populations sorted from spleen used in Figure 2B, i. Cells were sorted into IgD^hi^ (D1 70.1% of all B cells) follicular B (FOB) cells and IgD^lo^ (D2 21.1% of all B cells). D2 was further sorted into CD24^hi^CD43^+ve^ (D3 30.1% of D2) splenic B1 cells. Purity checks can be seen in (ii) and CD19 expression of sorted cells (iii) with FOB shown in black and B1a shown in red. **(C)** Example genotyping of TIM1^−/−^ BALB/c (i) and TIM1^−/−^ C57BL/6 (ii) mice used in Figure 2C. Wild-type (WT) mice show a 264-bp band whereas TIM1^−/−^ mice show a 383-bp band. **(D)** WT C57BL/6 and TIM1^−/−^ C57BL/6 B cells (IgD^lo^IgM^hi^CD21^hi^) were FACS sorted and cultured with (black bars) and without (patterned bars) apoptotic cells. Cultures were stimulated with R848 (i), CpG (ii), lipopolysaccharide (LPS) (iii), and OVA plus OVA-specific T cells (iv) and IL-10 measured after 72 h. Results are pooled from five mice. **(E)** Histogram plots of B cell markers in isolated B cell populations. Isotype control is shown in gray, WT BALB/c dotted black line, TIM1^−/−^ BALB/c dashed black line. Data representative of *n* = 4 (using 11 mice total). **(F)** FACs sorted splenic FOB (IgD^hi^IgM^lo^), MZB (IgD^lo^IgM^hi^CD21^hi^) and CD19^+ve^ peritoneal cavity (PerC) cells were cultured for 2 h with apoptotic cells in a 1:5 ratio. WT BALB/c is shown with solid bar, TIM1^−/−^ BALB/c with patterned bar. Data representative of two experiments (using nine mice total). **(G)** WT BALB/c and TIM1^−/−^ BALB/c B cells (IgD^lo^IgM^hi^CD21^hi^) were cultured with (black bars) and without (patterned bars) apoptotic cells and activated with CpG-B (i), and LPS (ii). IL-10 in the culture supernatants was assessed after 72 h. (*n* = 8). **(H)** Apoptotic cells were injected at the time of OVA-complete Freund’s adjuvant (CFA) antigenic challenge *in vivo* into WT BALB/c and TIM1^−/−^ BALB/c mice. Spleens were removed on D7 and restimulated with OVA peptide. IL-10 was measured in culture supernatants after 72 h (*n* = 3). **(I)** Splenocytes (taken from OVA-CFA antigenic challenged mice that had been given apoptotic cells) were labeled with CFSE and restimulated with OVA. Proliferation of OVA-specific T cells was measured. CFSE MFI of CD3^+ve^ T cells was determined and plotted as a % decrease from Day 1. WT spleen open circles, TIM1^−/−^ spleen filled circles. Data representative of two experiments with duplicate treatments per experiment. **(J)** Histogram showing representative screening data for CD21^−/−^ mice. Binding of anti CD21 antibody to WT B cells can be seen (dashed line) compared to no binding seen with CD21^−/−^ mice (solid line). Data representative of *n* = 6. **(K)** CD19^+ve^IgD^lo^IgM^hi^ B cells were FACs sorted from CR2^−/−^ and control C57BL/6 mice. Cells were cultured with (solid black bars) and without (patterned bars) apoptotic cells in the presence of CpG-B. IL-10 was measured in the culture supernatants after 72 h. Data are representative of two experiments using 10 mice in total. **(L)** CD19^+ve^IgD^lo^IgM^hi^CD21^hi^ B cells were FACs sorted from C1q^−/−^ and control WT C57BL/6 mice. Cells were cultured with (solid black bars) and without (patterned bars) apoptotic cells in the presence of R848 (i) or CPG (ii). IL-10 was measured in supernatants after 72 h. Representative of *n* = 4. **(M)** Splenocytes (from OVA-CFA challenged mice given apoptotic cells) were harvested on D7 and cultured with OVA. IL-10 (i) and IL-6 (ii) was measured in the supernatants after 72 h. WT controls are shown in open circles, C1q^−/−^ mice in filled circles. Data pooled from three individual mice, representative of three individual experiments using eight mice total. **(N)** Spleen CD19^+ve^ B cells were harvested from naive C57BL/6 and C1q^−/−^ mice. Cells were stained to determine proportion of B1 (IgD^lo^IgM^hi^CD21^hi^CD43^+ve^) B cells. WT are shown with open circles and C1q^−/−^ with filled circles. *N* = 6 mice. Statistical differences were determine by unpaired Student’s *t*-test **P* < 0.05, ***P* < 0.01, and ****P* < 0.001.

Figure S3 **(A)** Representative dot plots showing interaction of B cells and apoptotic cells (i) showing splenic follicular B cells on left and peritoneal cavity B1a cells on right. Upper plots show 4 h culture, lower 24 h culture. Single color control images for microscopy staining shown in Figure 3A, ii, can be seen in [**(A)**, ii]. Scale bar represents 20 μm. **(B)** Histogram plots for surface markers of IL-10^+ve^ B cells (in red), used to generate hybridomas in Figure 3D, compared to IL-10^−ve^ B cells (in black) and isotype control (in shaded gray). **(C)** Schematic representation of hybridoma generating process. **(D)** Supernatants from hybridoma clones (Figure 3D) and subsequent pooled supernatants used in Figure 4A were incubated with apoptotic cells. Binding could be seen in IL-10^+ve^ sups (dashed line) but not IL-10^−ve^ sups (solid lines) to jurkats (i) and thymocytes (ii) but not viable jurkats (iii).

Figure S4 **(A)** Supernatants used in Figure 4A showed no binding to viable jurkats. Cell membrane red, IgM green, DAPI blue. Scale bar represents 20 μm. **(B)** Single stained controls for Figure 4A showing single cell mask plasma membrane dye in red, IgM staining in green, and DAPI in blue. Scale bar represents 60 μm.
