# Supplementary figures and images for: Immune Tolerance to Apoptotic Self Is Mediated Primarily by Regulatory B1a Cells
Source: Front Immunol. 2018 Jan 19;8:1952. doi: 10.3389/fimmu.2017.01952 (PMC5780629; doi:10.3389/fimmu.2017.01952)

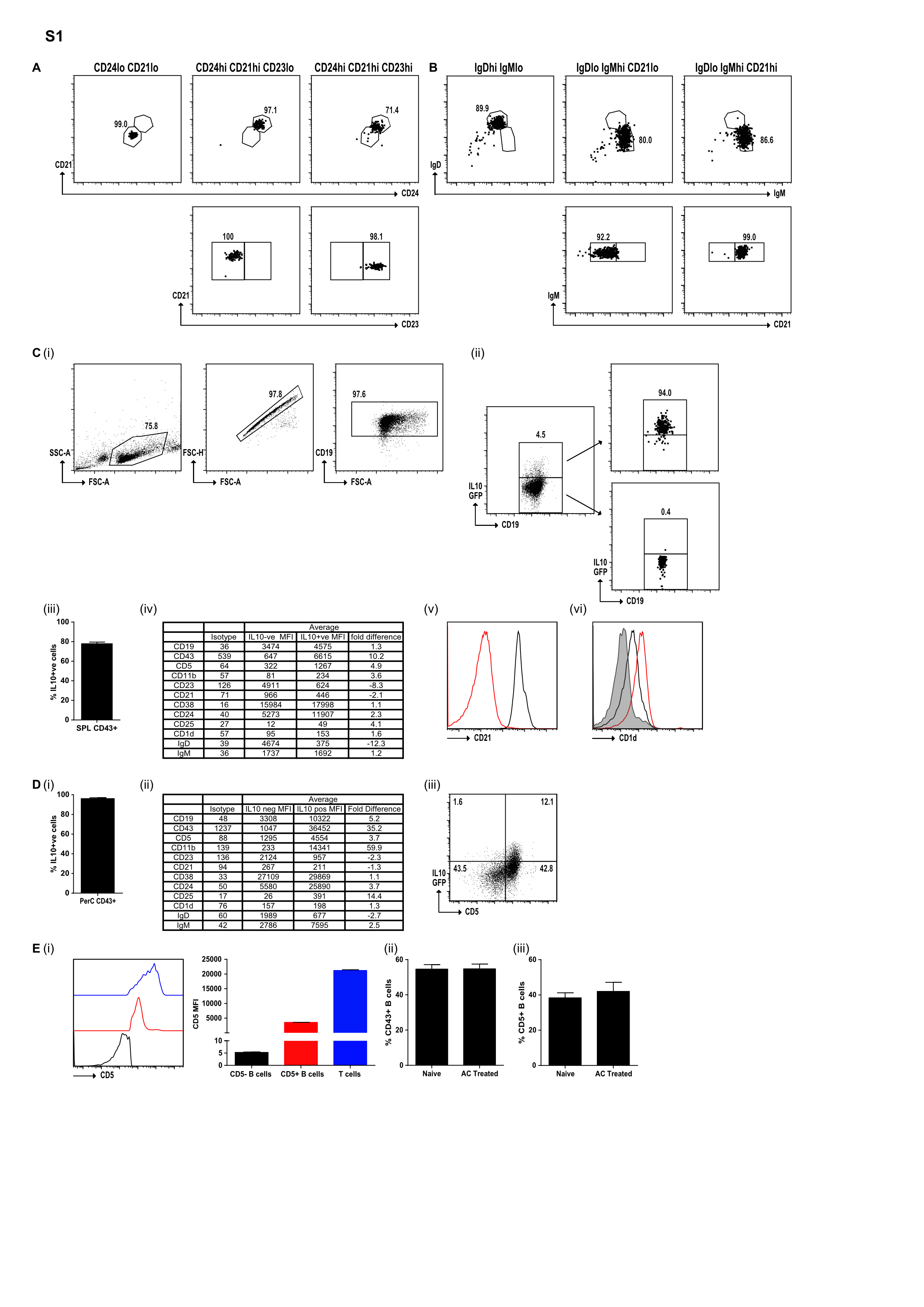

Supplement: Figure S1 — (A) Purity checks of populations A1, A3, and A4 used in Figure 1A. (B) Purity checks of populations C1, C3, and C4 used in Figure 1B. (C) Sorting strategy (i) and purity checks (ii) of splenic IL-10+ve cells which were further analyzed in Figure 1C. (iii) Percentage of IL-10+ve splenic CD19+ve B cells which express CD43 (as a marker of splenic B1a) is 77.8%. (iv) The average mean florescence intensity (MFI) data represented in Figure 1C (ii) are shown along with the mean fold difference. (v) Change in CD21 expression postculture is shown as a histogram with unstimulated marginal zone B cells shown in black and the same cells shown after 72 h R848 culture in red. (vi) Change in CD1d expression postculture is shown as a histogram with unstimulated CD19+ve cells shown in black and the same cells shown after 72 h R848 culture in red. Isotype control is shown in shaded gray. (D) (i) Percentage of IL-10+ve peritoneal cavity (PerC) CD19+ve B cells which express CD43 is 96%. (ii) The average MFI data represented in Figure 1E, ii, are shown along with the mean fold difference. (iii) Representative dot plot of peritoneal CD19+ve B cell used in Figure 1E, ii, showing expression of CD5 and IL-10. (E) (i) Level of expression of CD5 in peritoneal CD5−ve B cells (black), CD5+ve B cells (red) and T cells (blue). Percentage of CD43+ve (ii) or CD5+ve (iii) PerC B cells in Naiïve or apoptotic cellAC-treated mice used in Figure 1E and (S1D). [file Image_1.jpeg]

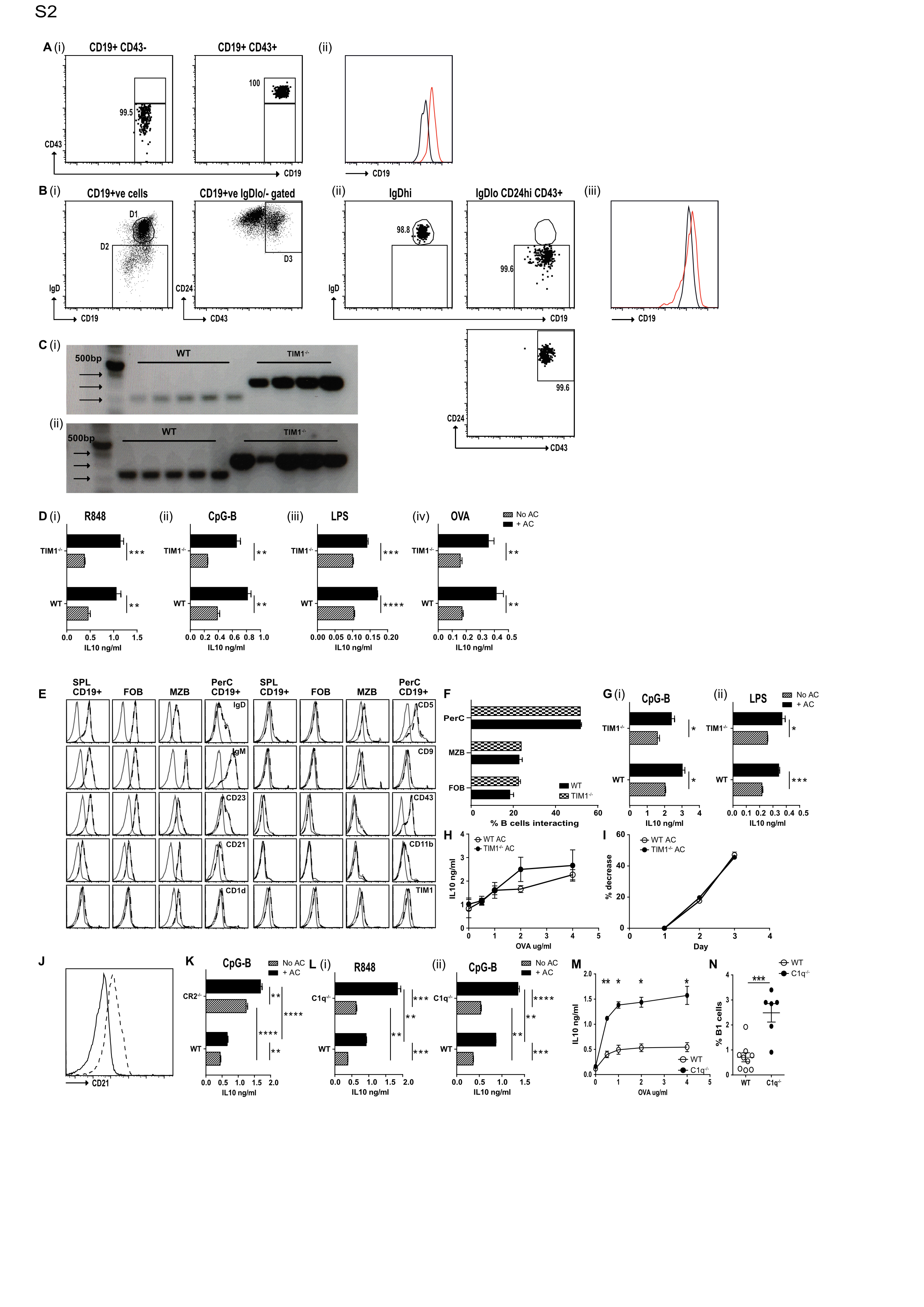

Supplement: Figure S2 — (A) Purity checks of peritoneal cavity (PerC) CD43−ve and CD43+ve (i) and CD19 expression of sorted populations with CD43−ve in black and CD43+ve in red (ii) used in Figure 2A. (B) Gating strategy of populations sorted from spleen used in Figure 2B, i. Cells were sorted into IgDhi (D1 70.1% of all B cells) follicular B (FOB) cells and IgDlo (D2 21.1% of all B cells). D2 was further sorted into CD24hiCD43+ve (D3 30.1% of D2) splenic B1 cells. Purity checks can be seen in (ii) and CD19 expression of sorted cells (iii) with FOB shown in black and B1a shown in red. (C) Example genotyping of TIM1−/− BALB/c (i) and TIM1−/− C57BL/6 (ii) mice used in Figure 2C. Wild-type (WT) mice show a 264-bp band whereas TIM1−/− mice show a 383-bp band. (D) WT C57BL/6 and TIM1−/− C57BL/6 B cells (IgDloIgMhiCD21hi) were FACS sorted and cultured with (black bars) and without (patterned bars) apoptotic cells. Cultures were stimulated with R848 (i), CpG (ii), lipopolysaccharide (LPS) (iii), and OVA plus OVA-specific T cells (iv) and IL-10 measured after 72 h. Results are pooled from five mice. (E) Histogram plots of B cell markers in isolated B cell populations. Isotype control is shown in gray, WT BALB/c dotted black line, TIM1−/− BALB/c dashed black line. Data representative of n = 4 (using 11 mice total). (F) FACs sorted splenic FOB (IgDhiIgMlo), MZB (IgDloIgMhiCD21hi) and CD19+ve peritoneal cavity (PerC) cells were cultured for 2 h with apoptotic cells in a 1:5 ratio. WT BALB/c is shown with solid bar, TIM1−/− BALB/c with patterned bar. Data representative of two experiments (using nine mice total). (G) WT BALB/c and TIM1−/− BALB/c B cells (IgDloIgMhiCD21hi) were cultured with (black bars) and without (patterned bars) apoptotic cells and activated with CpG-B (i), and LPS (ii). IL-10 in the culture supernatants was assessed after 72 h. (n = 8). (H) Apoptotic cells were injected at the time of OVA-complete Freund’s adjuvant (CFA) antigenic challenge in vivo into WT BALB/c and TIM1−/− BALB/ [file Image_2.jpeg]

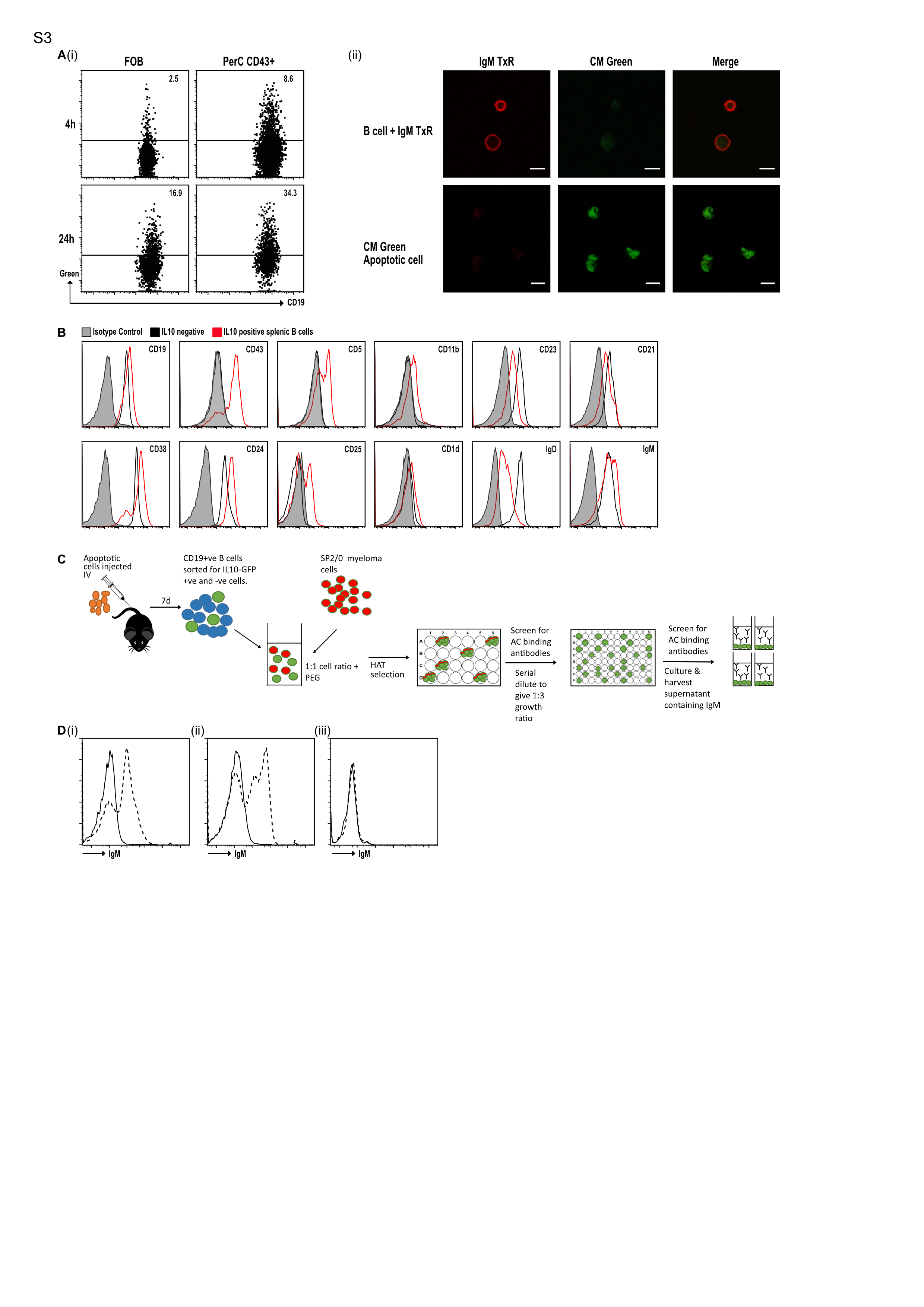

Supplement: Figure S3 — (A) Representative dot plots showing interaction of B cells and apoptotic cells (i) showing splenic follicular B cells on left and peritoneal cavity B1a cells on right. Upper plots show 4 h culture, lower 24 h culture. Single color control images for microscopy staining shown in Figure 3A, ii, can be seen in [(A), ii]. Scale bar represents 20 μm. (B) Histogram plots for surface markers of IL-10+ve B cells (in red), used to generate hybridomas in Figure 3D, compared to IL-10−ve B cells (in black) and isotype control (in shaded gray). (C) Schematic representation of hybridoma generating process. (D) Supernatants from hybridoma clones (Figure 3D) and subsequent pooled supernatants used in Figure 4A were incubated with apoptotic cells. Binding could be seen in IL-10+ve sups (dashed line) but not IL-10−ve sups (solid lines) to jurkats (i) and thymocytes (ii) but not viable jurkats (iii). [file Image_3.jpeg]

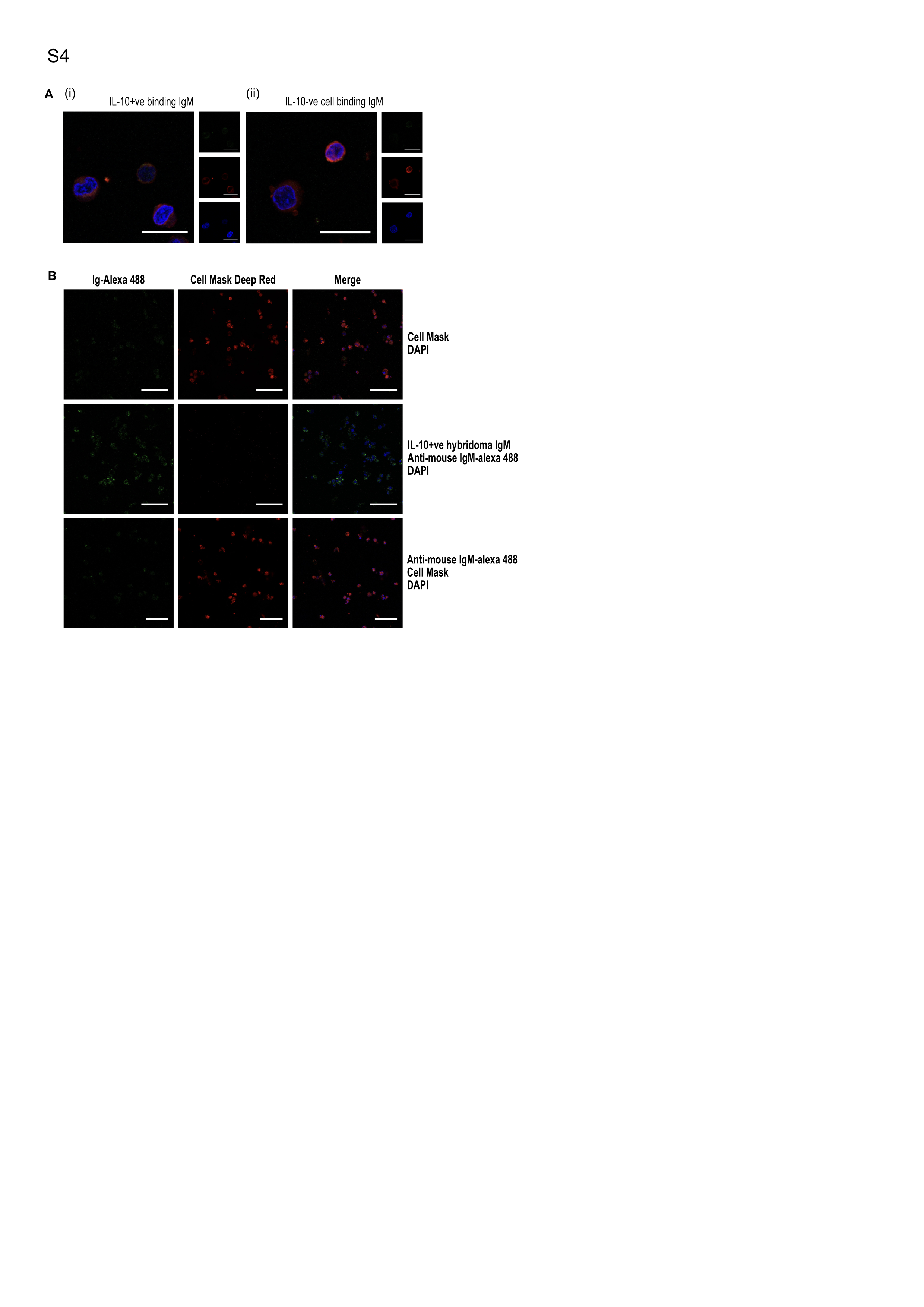

Supplement: Figure S4 — (A) Supernatants used in Figure 4A showed no binding to viable jurkats. Cell membrane red, IgM green, DAPI blue. Scale bar represents 20 μm. (B) Single stained controls for Figure 4A showing single cell mask plasma membrane dye in red, IgM staining in green, and DAPI in blue. Scale bar represents 60 μm. [file Image_4.jpeg]
